# Defining the integrated neighbourhood model: a systematic review of key domains and framework development

**DOI:** 10.1186/s12889-025-22582-x

**Published:** 2025-04-12

**Authors:** Fahad M. Iqbal, Seher Kayikci, Hayley Lowther-Payne, Mohamed Aly, Alan Askari, Rachel Wells, Afsana Bhuiya

**Affiliations:** 1 Imperial College London, St Mary’s Hospital, London, UK; 2 London Borough of Barnet, Colindale, NW9 4EW UK; 3 Applied Health Research Hub (Ahrh), University of Central Lancashire, Preston, Lancashire UK; 4 Brunel University, Kingston Lane, London, Uxbridge UB8 3PH UK; 5 North Central London ICB, Laycock PDC, Barnet, N1 1 UK

**Keywords:** Integrated health care systems, Community health services, Health policy, Health services research

## Abstract

**Background:**

Health systems are increasingly adopting Integrated Neighbourhoods (INs) to deliver hyper-local, community-based care that integrates health, social care, and public sector resources to address healthcare costs, improve outcomes, and reduce health inequalities. However, IN models lack a unified definition and standard framework for development and evaluation, limiting their scalability and effectiveness. This systematic review aims to establish a foundational framework for INs, identifying key domains to guide their implementation (including barriers of implementation, evaluation, and potential for future research.

**Methods:**

A systematic literature search, restricted to the English language, was performed to identify relevant studies with expert librarian support. Study quality was assessed with the Mixed-Methods Appraisal Tool (MMAT). A Braun and Clarke thematic analysis was conducted to identify recurring themes and extract key domains.

**Results:**

A total of 29 studies met the inclusion criteria, encompassing a diverse range of IN models with varying focus areas and methodologies. Seven key domains emerged as central to effective IN models: integrator host, integrator enablers, integrator partnership principles, core integrated workforce, core areas of work, and services provided. These domains support multidisciplinary collaboration, enhance resource utilisation, and promote community engagement. However, barriers such as funding limitations, digital exclusion, and inconsistent evaluation frameworks present challenges to IN scalability and sustainability.

**Conclusion:**

This proposed framework provides a starting point for a standardised structure for implementing and evaluating INs, guiding clinicians, academics, and policymakers in developing sustainable, equitable, and adaptable community-based care solutions with the potential to improve access to patients from low-socioeconomic and underserved communities.

**Trial Registration:**

PROSPERO ID: CRD42024597197; available: https://www.crd.york.ac.uk/prospero/display_record.php?RecordID=597197.

## Background

Health systems worldwide have increasingly turned to integrated care models to mitigate rising healthcare costs, improve patient outcomes, and reduce health disparities [1, 2]. One such model, Integrated Neighbourhoods (INs), focuses on delivering hyper-local, community-based care through collaboration across health, social care, and public sectors [3–6].

Despite widespread efforts, including various pilot programs across the UK and internationally [7], there remains no unified definition or standardised framework for IN development and operation. The Fuller Stocktake Report, aimed to provide a vision for integrated primary care, has been a strategic driver for INs and teams. It places primary care networks (PCNs) at the heart of IN development [8]. PCNs are a group of primary care practices that work together and are aligned to other health and social care staff and organisations, providing integrated services to their local population. A typical PCN patient population ranges from 30,000 to 50,000, though this is variable with some PCNs having lower or higher numbers, depending on their circumstances [9]. PCN-led INs, in particular, aim to address unmet health needs and reduce health inequalities, especially in the most deprived communities [3, 10]. The report further highlighted the need for clarity in IN structure, governance, and evaluation, but consensus remains elusive [8].

A similar approach can be seen in the concept of the Patient’s Medical Neighbourhood (PMN), which emphasises collaboration between primary care, other healthcare providers, and community services to improve patient outcomes, safety, and care coordination. The PMN model highlights the importance of clear role definitions, structured referral pathways, and leveraging technology to enhance communication, all of which align with the principles of INs [11]. These similarities underscore the growing recognition of place-based, integrated care models, but also highlight the need for a structured framework to guide implementation and evaluation.

The absence of a clear IN framework hampers efforts to assess the impact of INs on health and patient outcomes, resource utilisation, and health inequalities. Without a structured understanding of the key domains driving successful INs, replication and scaling of these models across health systems remain challenging.

As such, this systematic review seeks to address this gap by defining the IN model and proposing a comprehensive framework.

## Methods

### Study design and objectives

This systematic review was conducted in accordance with the Preferred Reporting Items for Systematic Review and Meta-Analysis (PRISMA) guidelines [12]. The review was registered at the International Prospective Register of Systematic Reviews (CRD42024597197).

This review sought:

1. To define the Integrated Neighbourhood (IN) model through a comprehensive review of existing literature and to characterise its application in real-world settings.
2. To identify reported barriers and enablers of implementing INs.
3. To develop a structured framework of domains that underpin the establishment, maturation, and supports evaluation of INs.

### Search strategy and databases

A systematic search, with expert librarian support, was performed using electronic databases through Ovid in Medline, EMBASE, Health Management Information Consortium (HMIC), CENTRAL, WHO International Clinical Trials Registry Platform (ICTRP), and PsycINFO databases. The appropriate MeSH terms and free text all field search was performed and combined with appropriate Boolean operators for “Integrated care”, “multidisciplinary team”, “multidisciplinary”, “multiprofessional”, “Integrated Neighbourhood”, “integrated wellness”, “Alliance”, “people-centred”, “cross-sector”, Communit*,“primary care”, “ICB”, “primary healthcare”, “neighbourhoods”, gp, “General Practice”, “local health*”, “Inequalit*”, governance, “outcome”, “development”, “implementation”, “evaluation”, “barrier”, “challenge”, “difficulties”, “evaluation”, “survey”, “Wellbeing”, “enhancement”, “influence”, “Impact”.

Further studies not captured by the search were identified through bibliometric cross-referencing. Moreover, grey literature and relevant international and UK websites, including Government and health organisations (e.g., World Health Organisation, Public Health England, NHS England, Department of Health and Social Care); think tanks and research institutes (e.g., King’s Fund) were searched.

All identified studies were uploaded to Covidence (Melbourne, Australia), a Cochrane supported systematic review package tool [13]. Initial screening was conducted by two investigators (FMI and MA) to determine if the eligibility criteria were met. Discrepancies were resolved by discussion. Studies meeting the inclusion criteria underwent full-text screening; supplemental references were scrutinised for additional relevant articles.

### Study selection criteria

For the purpose of this review, both integrated neighbourhoods and healthcare neighbourhoods were included in the selection criteria, as they represent complementary but distinct models of localised, community-based care.

Integrated Neighbourhoods encompass a broad, cross-sector approach that integrates health, social care, voluntary sectors, and public services to address social determinants of health, reduce health inequalities, and enhance community well-being. INs are characterised by their multi-agency collaboration, often involving local government, public health teams, PCNs, community organisations, and voluntary sector partners to deliver coordinated, holistic care.

In contrast, Healthcare Neighbourhoods (HNs) are more clinically focused, centering on the coordination of healthcare services within a defined geographical area to improve access, integration, and patient outcomes. HNs are typically anchored in PCNs or local health systems, working towards more efficient healthcare service delivery but often with less emphasis on social determinants and broader community engagement compared to INs.

While both models share common goals of improving population health through integration, the primary distinction lies in INs’ wider scope, which extends beyond healthcare to include social determinants, community development, and cross-sector partnerships. The review predominantly discusses INs, given their broader remit and their increasing adoption within UK health policy frameworks (e.g., the Fuller Report). However, studies focusing on HNs were included where they provided relevant insights into the integration of health services at the community level.

The last search was performed on 25 th October 2024, restricted to the English language. Filters were applied to remove abstracts, conference articles, opinion pieces, reviews, meta-analyses, and studies dating before 1990 in order to prioritise the most relevant texts. While we did not apply any geographic restriction to study inclusion, the majority of included studies were from the United Kingdom, we also identified and included relevant international studies from Canada, the USA, Germany, and Scotland. This distribution may reflect a stronger policy and academic focus on IN models within the UK context. However, reference lists of relevant meta-analyses and systematic reviews to identify any primary studies that may have been overlooked during our initial search was also undertaken. Studies with inadequate published data were additionally excluded. This was assessed by evaluating whether studies provided clear descriptions of their methodology, data collection, and findings. Studies were excluded if they lacked essential details necessary for thematic analysis or risk of bias assessment. This included cases where outcome measures were vaguely reported, methodology was insufficiently described, or key data were missing. Two independent reviewers (FMI and MA) conducted this assessment, and disagreements were resolved through discussion to ensure consistency in exclusion decisions.

### Data extraction and analysis

All included study characteristics were extracted independently by two investigators (FMI and MA) with consensus achieved. Disagreement between the two reviewers was resolved by discussion. All full text reports of studies identified as potentially eligible after title and abstract review were obtained for further review.

Outcome measures included in this review were those evaluating the impact of integrated or healthcare neighbourhoods on health and social outcomes, resource utilisation, community engagement, reduction of health inequalities, barriers and facilitators recognised during implementation.

Given the expected heterogeneity of the IN models, a narrative synthesis of the included studies was performed to summarise and interpret findings from the included studies, particularly when synthesising heterogeneous evidence that varied in methodology and context [14]. This involves an involves an iterative process of developing a preliminary synthesis, exploring relationships within and across studies, and assessing the robustness of the synthesis. This approach was used to contextualise key findings related to INs and their implementation.

Furthermore, a Braun and Clarke thematic analysis was undertaken in order to create a structured framework for evaluation [15]. employed an emergent coding strategy initially conducted by AB, with additional emergent codes refined and expanded by FMI and MA to ensure comprehensive theme identification. The process began with data familiarisation, where reviewers identified and uploaded relevant text into NVivo v.12. AB conducted the initial emergent coding, capturing themes that organically arose from the data. FMI and MA then reviewed the coding, refining, and expanding the thematic structure to ensure no significant insights were overlooked. Codes were iteratively grouped into higher-order themes, which were continuously reviewed and refined until thematic stability was achieved. Thematic saturation was assessed by tracking the emergence of new themes across studies, with NVivo’s query tools used to determine when no additional concepts were identified. A final validation process ensured thematic coherence, with discrepancies resolved through group discussion and cross-referencing findings with the original study data. This structured yet flexible approach enhances transparency and reproducibility, ensuring comprehensive theme capture while allowing for dynamic refinement by multiple reviewers.

### Quality assessment (risk of bias)

Risk of bias was done using the Mixed-Methods Appraisal Tool (MMAT). MMAT has been used in a number of systematic reviews, and can be utilised in assessing the quality of studies with various designs applicable in this study [16]. Quality assessment was assessed by one reviewer (FMI) and validated by a second (MA).

## Results

### Study characteristics

**Fig. 1:**
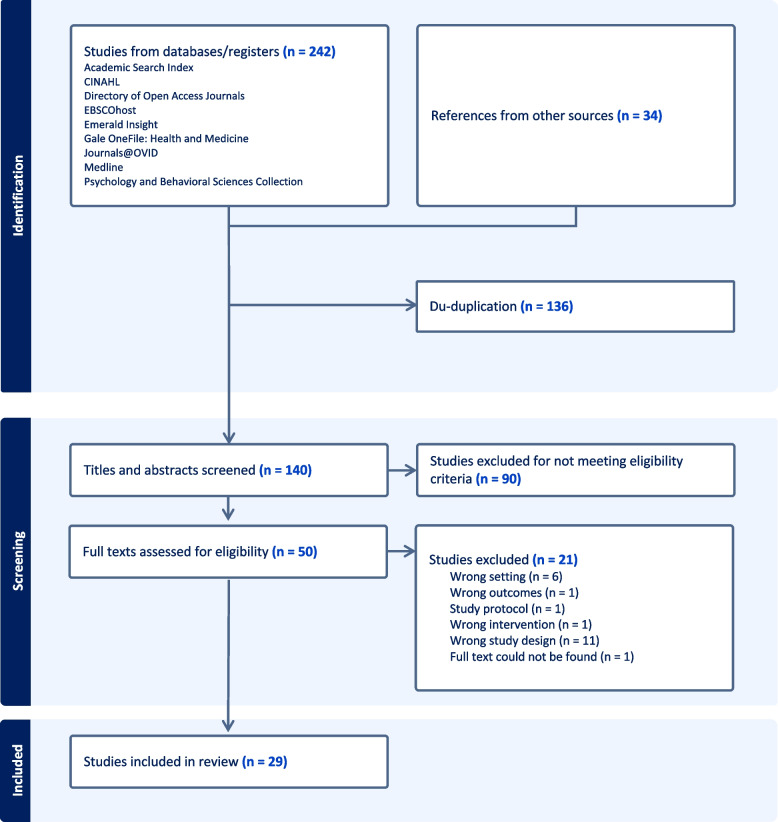
PRISMA flow diagram

A total of 276 results were retrieved through the literature search (Fig. 1). Upon deduplication and applying filters (as above), full text review was performed for 50 articles resulting in 29 articles included in our review. Studies were conducted primarily in the United Kingdom (*n* = 28).

Evaluation reports (*n* = 11) were the most common study design, followed by case studies (*n* = 8) and mixed methods (*n* = 6). The study characteristics are shown in Tables 1 and 2. A PRISMA flow diagram can be seen in Fig. 1.

**Table 1:** Study characteristics and included IN models of included studies

Author	Year	Country	Study Design	Sample Size	Population Characteristics	IN Model Description	Duration of IN Implementation	IN Leadership/Structure	Workforce Integration	Community Engagement	Key Domains
Bungay et al. [17]	2010	UK	Evaluation Report	N/A (review of different Arts on Prescription programs in the UK)	People with mental health issues (e.g., depression, anxiety, social isolation)	Arts on Prescription (AoP) schemes offering creative activities to support mental well-being	Ongoing programs, varied across different UK regions	Programs facilitated by artists, musicians, not therapists	Collaboration between health and social care, community artists, and musicians	Engagement through creative activities aimed at mental health recovery	Mental well-being, social inclusion, community support
Edmonton Community Partnership [18]	2022	UK	Evaluation Report	150 + participants	Includes a wide range of Edmonton residents, including young people, marginalised communities, people with disabilities, and mental health service users	Community Powered Edmonton, a partnership between the NHS, voluntary sector, and community, aiming to reduce health inequalities and improve services in Edmonton	3 months	Collaborative leadership from Edmonton Community Partnership, New Local, Healthwatch Enfield	Cross-sector collaboration between the NHS, local authority, and voluntary sector through workshops and community activities	Engagement through creative workshops, focus groups, and surveys, involving diverse community groups	Health inequalities, mental health, social determinants of health, community collaboration
Lent et al. [19]	2022	UK	Evaluation Report	Not applicable	Community groups, local councils, voluntary sector organisations	Community-powered health approach for prevention through partnership between the NHS, local authorities, and communities	Ongoing	Leadership from community, NHS, local authorities, voluntary sectors	Cross-sector collaboration between NHS, local authorities, and communities	Workshops, surveys, focus groups involving diverse community stakeholders	Social determinants of health, community power, prevention-based care
Health and Social Care Alliance Scotland (the ALLIANCE) [20]	2021	Scotland	Evaluation report	32 Local authorities/Health and Social Care Partnerships (HSCPs)	Marginalised groups, disabled people, people with long-term conditions, unpaid carers	Changes to social care delivery during COVID- 19, with a focus on emergency legislation and the use of flexible support models	Temporary, during the pandemic	Collaboration between local authorities, HSCPs, and third sector organisations	Teams worked flexibly, using hybrid models combining remote work with face-to-face services	Virtual services, digital inclusion initiatives, alternative care delivery models adopted during COVID- 19	Social care assessments, digital inclusion, emergency care packages
Dickerson et al. [21]	2024	UK	Mixed Methods	N/A	Diverse inner-city population with high levels of deprivation, including South Asian and White British communities	The Reducing Inequalities in Communities (RIC) programme focused on interventions targeting health inequalities and premature mortality in Bradford	5 years	Collaboration between NHS England, local authorities, and academic institutions (Born in Bradford, University of York)	Cross-sector collaboration between health, local authorities, and academic teams	Community engagement through focus groups, workshops, and evaluation of readiness for interventions	Health inequalities, premature mortality, community readiness for interventions
Such et al. [22]	2017	UK	Mixed Methods	70 survey respondents, 8 case studies	New migrant populations (refugees, asylum seekers, undocumented migrants)	Primary care practices adapting services for migrant populations, focused on equitable care	Not specified	Local leadership from general practices, NHS, and external agencies	Practices collaborated with local charities, community groups, and police	Engagement through patient involvement groups, interpreter services, and tailored outreach efforts	Equitable care, addressing trauma, culturally competent care, social determinants of health
South et al. [23]	2021	UK	Qualitative synthesis of case studies	24 case studies included	Focused on community hubs and green/blue space wellbeing projects, serving marginalised groups	Interventions to promote wellbeing and social relations through community infrastructure	Varied, with some projects ongoing	Collaboration between NHS, local authorities, and community organisations	Multidisciplinary teams worked with community members to design and deliver services	Community participation through co-production, participatory learning, and decision-making	Wellbeing, social capital, community infrastructure, empowerment
LGA [24]	2020	UK	Evaluation Report	N/A	Local government, health services, and community organisations	Asset-based approach to community health and wellbeing, with citizens as co-producers of health	10 years	Local government and public health leadership with a community-centred approach	Collaborative work between local government, public health teams, and community organisations	Promoted through networks, relationships, and mutual support, empowering communities to take control of their health	Asset-based approaches, community empowerment, mutual aid, social capital
Merron Simpson, Royal College of General Practitioners, Health Creation Alliance [25]	2021	UK	Evaluation Report	N/A	General practice and primary care networks, local communities, local authorities	PCNs as a vehicle for place-based working to reduce health inequalities through partnerships with communities and local partners	Ongoing	Leadership through PCNs in collaboration with local partners such as voluntary and community sectors	Integration between primary care, local authorities, and voluntary sectors	Community-led initiatives and strategic partnership development	Addressing health inequalities, mental health, social capital, community empowerment
Public Health Wales [26]	2023	Wales	Qualitative case study synthesis	14 case studies	Includes older adults, individuals with mental health issues, caregivers, and marginalised populations	Social prescribing models connecting individuals to community assets for health and well-being support	Varies depending on case study	Leadership from social prescribing practitioners, local organisations, and NHS integration	Interdisciplinary teams of social prescribing practitioners, local authorities, and community organisations	Engagement through personalised"what matters"conversations, connections to local groups, and follow-up support	Community-based care, mental health, loneliness, financial advice, caregiver support
East Midlands Academic Health Science Network [27]	2019	UK	Survey and qualitative case studies	57 survey responses, 5 case studies	General population across East Midlands with focus on socially deprived, isolated, or vulnerable populations	Social prescribing models varied by county, connecting patients to non-clinical services and community groups	Varied across regions, most models in operation less than a year	Leadership from local councils, CCGs, voluntary sector organisations, PCNs	Integrated teams, including link workers, GPs, and community organisations	Engagement via personalised support, linking patients to social, emotional, and practical services	Health inequalities, social isolation, mental well-being
Tull [28]	2019	UK	Policy guidance	Not applicable	Populations showing vaccine hesitancy; settings include LMICs, HICs	Vaccine hesitancy, despite availability of vaccines, includes refusal or delay of vaccines despite availability of vaccination services	Varies by country	Leadership from government stakeholders, healthcare providers, NGOs	Integration between healthcare providers and policy makers to address misinformation	Community engagement through tailored communication strategies, use of religious/community leaders	Health inequalities, vaccine confidence, communication strategies
Kassianos et al. [29]	2015	UK	Qualitative study	16 interviews	Healthcare professionals including GPs, geriatric specialists, psychiatry consultants, diabetes specialists, social workers, and practice managers in North West London	Integrated Care Pilot focusing on multidisciplinary group meetings as part of care planning and coordination for patients with diabetes and the elderly	1 year (initial evaluation period)	Collaborative leadership involving NHS trusts and other organisations with multidisciplinary meetings	Involvement of various healthcare providers, including GPs, consultants, and community services, in structured meetings	Not a primary focus in this study. Engagement was professional-centred rather than community-based	Integration between primary and secondary care, shared learning among professionals, and holistic patient care strategies
Change Starts With Me Evaluation Team [30]	2019	UK	Mixed Methods Evaluation	Two cohorts (14 and 16 participants)	GPs, practice nurses, practice managers, and pharmacists in Primary Care Networks in South East England	Multi-professional leadership programme focusing on system improvement within PCNs	8 months	Collaborative leadership involving PCN teams and clinical commissioning groups	Multi-disciplinary collaboration among GPs, nurses, managers, and pharmacists	Peer consulting, co-created service improvement projects, and community-engaged initiatives	Collective leadership, transformation, workforce development, community-focused care
Public Health England [31]	2019	UK	Policy report	11 local authorities involved in co-production and testing	Local populations with high obesity rates; stakeholders included local councils, public health, and community members	Whole systems approach to obesity co-produced with local authorities and tested for implementation across pilot and test local authorities	4 years	Leadership through local councils and PHE with support from community stakeholders	Multi-sector collaboration between local authorities, public health teams, and community partners	Community engagement was central, with stakeholders involved in workshops, system mapping, and action planning	Obesity prevention, system mapping, health inequalities, multi-sector collaboration
Future Spaces Foundation [32]	2019	UK	Case study and expert panel discussion	Not applicable	Includes urban populations, focusing on city dwellers across various demographics (seniors, disabled individuals, migrants)	Explores how urban design and built environments contribute to loneliness and opportunities for social connection	No specific timeline	Leadership provided by the Future Spaces Foundation in collaboration with architects, social scientists, and policymakers	Multidisciplinary collaboration involving urban designers, architects, and public health professionals	Focus on reshaping urban environments (housing, public spaces, and transport infrastructure) to promote social connections	Urban loneliness, housing design, public spaces, social interaction, inclusive design
Cheetham et al. [33]	2018	UK	Mixed-methods study (qualitative and quantitative)	72 participants (25 service users, 23 non-users, 14 group service users, 9 staff members)	Targeted individuals in deprived communities with complex health and social needs	Live Well Gateshead (LWG) integrated wellness service addressing multiple social determinants of health	2 years	Local authority and NHS integration led by multidisciplinary wellness coaches and community teams	Interdisciplinary teams of wellness coaches, community capacity building staff, and NHS workers	Engagement via workshops, group sessions, and 1:1 tailored support	Health and wellbeing, community capacity building, social isolation reduction
Reynolds [34]	2018	UK	Ethnographic study	2 sites (urban and rural communities)	Disadvantaged communities in England (Westin Hill and Craybourne)	Area-based empowerment initiative to improve local communities through resident-led engagement	10 years	Leadership by local residents, supported by Local Trust, with decision-making committees	Multidisciplinary collaboration between residents, local authorities, and community workers	Community engagement through consultation, resident participation, and decision-making committees	Community engagement, empowerment, boundary work, participation
Orton et al. [35]	2017	UK	Systems evaluation and ethnographic fieldwork	10 sites, 138 interviews, focus groups and 440 h of observation	Residents from 150 disadvantaged areas in England	Area-based empowerment initiative (Big Local), focused on resident-led, collective action to improve local areas	10 years	Leadership by local residents, supported by Local Trust and delivery partners	Multidisciplinary teams, including residents, council workers, and external partners	Engagement through resident-led initiatives, workshops, events, and decision-making meetings	Community empowerment, social context, collective action, system co-evolution
Findlay et al. [36]	2017	UK	Qualitative case study and framework development	N/A	Focused on disadvantaged communities across London and pilot sites in suburban/rural areas	Whole systems approach engaging communities and local organisations to improve health and well-being	Ongoing since 2007	Leadership provided by local authorities, community members, and public health organisations	Cross-sector collaboration involving public, private, and third sectors with multidisciplinary teams	Engagement through community-driven action plans, workshops, and volunteering	Community well-being, resilience, empowerment, health inequalities
Ejbye et al. [37]	2016	UK	Case study synthesis	5 partner sites, specific sample size varies across sites	Includes people living with HIV, people recovering from cancer, individuals with mental health issues, and those in marginalised communities	Person- and community-centred approaches for health and wellbeing across five local sites, including peer support, self-management education, health coaching, and asset-based approaches	18 months	Local leadership through community organisations, NHS, and voluntary sector organisations	Multidisciplinary teams worked across sectors with peer support and health coaching programs	Engagement through peer support, group activities, and workshops involving local communities	Peer support, self-management, health coaching, community-centred approaches
Groene et al. [38]	2016	Germany	Case study	71,000 inhabitants enrolled in Gesundes Kinzigtal	Focused on individuals with chronic diseases and a broad population-based approach	Integrated care model (Gesundes Kinzigtal) targeting the Triple Aim (better population health, better care experience, lower per capita costs)	Ongoing since 2006	Leadership through a regional integrator and collaboration with sickness funds	Multidisciplinary care teams across health and social care providers	Community engagement through shared decision-making, health education programs, and self-management support	Population health management, patient engagement, integrated care
Farmer et al. [39]	2014	Scotland	Community-based participatory action research (CBPAR)	Four Scottish communities (rural and remote), population size < 3,000	Residents of rural communities with high social capital, focused on primary healthcare services	Community-based participatory design for healthcare services based on local priorities and needs	2008–2010	Community-led leadership with NHS Highland support	Multidisciplinary collaboration involving local practitioners and community members	Engagement through workshops, interviews, and planning exercises with local residents	Primary healthcare, emergency care, anticipatory care, wellbeing improvement
Ben Collins, The King’s Fund [40]	2015	USA (Alaska)	Case study synthesis	65,000 Alaska Native people served	Alaska Native people with significant health disparities served by an integrated, community-led health system	Southcentral Foundation’s ‘Nuka’ model of care – a relationship-based, integrated health system serving Alaska Native people	Ongoing since 1998	Leadership provided by Alaska Native people themselves, with customer-owners driving the design and management of care	Multidisciplinary teams consisting of primary care, mental health, dental, and traditional healing services	Community engagement through customer-owner feedback, involvement in decision-making processes, and co-production of care plans	Integrated care, community-driven leadership, holistic wellness, relationship-based care
NIHR [41]	2024	UK	Case study review	Pilot in Northwest London	General population in socially deprived areas	CHWWs support health promotion, chronic disease management, and social care navigation	Initial pilot phase with plans to expand nationally	Pilot leadership provided by Westminster community services and CHWWs integrated with GP practices	CHWWs collaborate with GPs, community services, and local authorities	Active community engagement through home visits, workshops, and one-on-one support	Health promotion, prevention, social care integration
NHS Confederation [42]	2022	UK	Case study	Not specified	General population in York, with a focus on socially isolated and lonely individuals	Asset-based community development model aimed at reducing loneliness and isolation	Ongoing since 2015	Leadership provided by City of York Council, collaboration with health and voluntary sectors	LAC (Local Area Coordination) teams work closely with GPs, social services, and community groups	Community engagement through LACs, volunteering, and initiatives like walking groups	Reducing loneliness, social isolation, non-clinical GP visits
Dale McMurchy Consulting [43]	2022	Canada	Case study analysis	407,000 clients served by Community Health Centres (CHCs)	Equity-seeking populations with higher primary care needs, facing barriers to care	Community Health Centres (CHCs) provide comprehensive interprofessional primary healthcare and social services to underserved communities	Ongoing	CHCs led by interprofessional teams with leadership from the community and public health sectors	Interprofessional teams including social workers, physicians, and community health workers	Engagement through social care services and health promotion activities that address the social determinants of health	Primary care, social care, health promotion, equity
NHS England [44]	2023	UK	Case study	28,595 patients	Population with significant mental health issues and high unemployment in a deprived seaside town	Integrated Neighbourhood Team (INT) including GP practices, pharmacies, mental health, and social services	10 years	Local PCN leadership, working with local authority, schools, charities, and faith groups	Multi-agency workforce including Occupational Therapists, Psychological Therapists, Mental Health Professionals, and local charities	Community engagement through youth hub, local charities, and schools	Youth mental health, employment, education, mental health service access
Institute of Health Equity [45]	2020	UK	Review and analysis of health equity	Not applicable	Population of England, with a focus on health inequalities across socioeconomic groups	Analysis of health inequalities and social determinants of health	Ongoing since 2010	Leadership from public health and academic experts with recommendations for government action	Not directly applicable	Community empowerment was one of the key features in the original Marmot Review	Health inequalities, social determinants of health, austerity

**Table 2:** Evaluation and impact of IN models

Study	Evaluation Approach	Outcome Measures	Evaluation Frameworks	Key Findings	Impact on Health Inequalities	Impact on Health Outcomes	Impact on Resource Utilisation	Barriers to Implementation	Facilitators of Implementation	Conclusions
Bungay et al. [17]	Mixed qualitative and quantitative (using Warwick-Edinburgh Mental Well-Being Scale, qualitative feedback from participants)	Mental well-being, social inclusion, community engagement	Use of the Warwick-Edinburgh Mental Well-Being Scale (WeMWBS)	Participation in creative activities raised self-esteem, enhanced social skills, and improved mental well-being	Focused on socially isolated individuals; positive impacts on mental health and community integration	Improved mental well-being, social inclusion	Not directly measured, but discussed in the context of reducing antidepressant use and primary care strain	Lack of a formal evidence base for effectiveness, resource constraints, institutional barriers	Enthusiasm from local GPs and community practitioners, engagement with voluntary sectors	AoP can enhance mental well-being and social inclusion; further robust evaluations are needed to demonstrate cost-effectiveness
Edmonton Community Partnership [18]	Qualitative feedback from workshops, focus groups, and surveys	Mental well-being, trust in services, community collaboration	No formal evaluation tool specified	Addressed health inequalities, built trust between services and communities, strengthened local voluntary sector, identified key issues like safety, mental health, and social isolation	Positive impacts on health inequalities through improved engagement and understanding of community needs	Improved community collaboration and mental well-being through engagement with local services	Not directly assessed, but improved trust and collaboration expected to reduce strain on public services	Lack of trust in services, language barriers, digital exclusion, lack of youth provision, poverty	Strong partnership between NHS, voluntary sector, and community groups, focus on collaboration and local assets	Trust-building, ongoing collaboration needed to address health inequalities and improve services
Lent et al. [19]	Qualitative feedback from community participants, evidence submissions	Engagement, community empowerment, prevention, health outcomes	No formal evaluation tool specified	Promotes a paradigm shift toward a prevention-based, community-driven healthcare model for tackling inequalities	Positive impacts on health inequalities and engagement, particularly in deprived areas	Improved community engagement, trust-building, and collaboration among health and social care sectors	Not directly assessed but expected to reduce demand on acute services	Resistance to cultural change within the NHS, lack of sustainable funding for community-driven models	Strong community leadership and collaboration between sectors, commitment from national bodies for prevention-focused policies	Advocates for a long-term moratorium on structural reform, shift toward prevention through community partnerships, and more community engagement in strategic decision-making
Health and Social Care Alliance Scotland (the ALLIANCE) [20]	Desk-based research, correspondence with local authorities and HSCPs, review of guidance documents	Changes to service delivery, digital inclusion, community engagement, access to care	No formal tools specified	Highlighted issues with digital exclusion, reduced social care services, and flexibility in service provision	Positive impact on access to care for those able to use digital services; negative impact on those facing digital exclusion	Improved flexibility of service delivery, but reduced in-person support created challenges	Resources stretched due to emergency legislation and workforce limitations, especially during high peaks of the pandemic	Strong collaboration between sectors, rapid digital transformation, and emergency funding supported flexible responses	Recommendations for future policy changes to sustain flexible services, address digital inequalities, and improve transparency in decision-making	need to address digital exclusion and sustain improvements in flexibility and collaboration between sectors in the post-pandemic period
Dickerson et al. [21]	Qualitative analysis of feedback from focus groups, use of the Community Readiness Model (CRM)	Reduced hospital admissions, improved financial benefits through welfare advice, improved community engagement	No formal evaluation framework specified	Reduced unplanned hospital admissions through targeted interventions (CLICS, PaCT), improved financial well-being through welfare advice services	Positive impact on reducing health inequalities through integrated care pathways and support services	Improved health outcomes through reduced unplanned hospital admissions and enhanced community engagement	Reduced hospital admissions and A&E visits, but no direct assessment of cost-effectiveness or resource utilisation reported	Funding constraints, digital exclusion, challenges in community readiness and engagement	Strong collaboration between sectors, targeted interventions through integrated care pathways, use of big data (Connected Bradford)	Interventions targeted at reducing inequalities were effective, but future programmes should integrate more robust evaluations from the start, with a focus on community engagement
Such et al. [22]	Survey with descriptive statistical analysis and thematic analysis of case studies	Adaptations to services (screening, vaccination, health checks, interpreter use, outreach)	No formal evaluation frameworks specified	Practices adapted to address wider social determinants, trauma, and culturally competent care despite resource constraints	Significant impact on improving access to primary care for marginalised migrant groups	Improved delivery of culturally competent care and enhanced engagement with migrant populations	No direct resource utilisation measures, but concerns about capacity, workload, and burnout	Lack of funding and secure resources, staff burnout, unclear guidelines on migrant healthcare entitlements	Strong practitioner commitment to equitable care, collaboration with external agencies and community organisations	Primary care services are adapting to migrant needs despite resource constraints; more formal evaluation is needed to assess long-term outcomes
South et al. [23] and Curry et al. [46]	Framework analysis of case study findings	Outcomes at individual, community, and organisational levels (e.g., social inclusion, confidence building, community empowerment)	No formal evaluation framework specified	Case studies highlighted the importance of spaces for social interaction, partnerships, and long-term funding in improving community wellbeing	Positive impact on addressing social inequalities and promoting community engagement	Improved social relations, increased confidence, and community empowerment	Not directly assessed, but improved service delivery and collaboration were highlighted	Challenges in securing long-term funding, digital exclusion, difficulties in evaluating diverse activities	Strong partnerships, long-term funding, volunteer engagement, and co-production with communities	Practice-based case studies provide valuable insights into community wellbeing initiatives; further work needed to develop robust synthesis methods
LGA [24]	Qualitative insights from practitioners and experts	Community engagement, resilience, empowerment, social support	No formal evaluation frameworks specified	Asset-based approaches have empowered communities, improved social capital, and fostered partnerships but face challenges in scaling up at a system level	Addressed social inequalities by empowering communities, but structural barriers remain	Improved social cohesion, mutual support, and resilience in communities	No direct resource utilisation analysis, but highlighted the need for sustainable funding	Short-term funding, structural barriers, challenges in changing professional mindsets	Strong leadership, long-term partnerships, and community ownership of programs	Asset-based working has been embraced across the UK but needs further scaling and systemic support for long-term sustainability
Merron Simpson, Royal College of General Practitioners, Health Creation Alliance [25]	Qualitative thematic analysis from interviews with healthcare professionals and local partners	Improved relationships between primary care and community partners, enhanced community engagement	No formal evaluation framework specified	PCNs showed potential in addressing health inequalities through community-led initiatives, but challenges in capacity and systemic tensions remain	Positive impact on addressing health inequalities, especially in communities with low levels of social capital	Improved community engagement and partnership development, reducing strain on primary care services	Not directly assessed but positive outcomes expected in reduced demand for healthcare services	Capacity issues, lack of strategic roles within PCNs, limited incentives in the DES Contract	Strong collaboration between PCNs, local authorities, and community organisations; use of community engagement tools	Primary care networks must enhance collaboration with communities and develop new pathways to address health inequalities effectively
Public Health Wales [26]	Qualitative case studies assessing social prescribing pathways and impacts	Improved mental health, social engagement, physical well-being, financial stability	No formal evaluation framework; outcomes based on qualitative feedback	Positive improvements in mental health, physical health, and social well-being for case study participants	Addressed social isolation, financial stress, and mental health challenges in marginalised populations	Improved mental health, social connectedness, and financial well-being for participants	Not directly assessed but reported improvement in service delivery through better engagement	Limited funding, digital exclusion, and reluctance to engage with services due to stigma	Strong relationships between social prescribing practitioners and community groups, flexible and personalised support	Effective for addressing diverse health and well-being needs; further integration and resources are needed to scale up social prescribing in Wales
East Midlands Academic Health Science Network [27]	Survey analysis and qualitative thematic analysis of case studies	Reduced GP attendance, improved mental health and social connections	No formal frameworks; evaluation based on self-reported outcomes	Improved patient outcomes, reduced GP attendance, increased social engagement and well-being	Positive effects on mental well-being and social isolation, especially among older adults and vulnerable groups	Improved mental health, reduced loneliness, improved social connections	Reduction in GP appointments and reliance on medical services	Inconsistent funding, lack of standardised evaluation, digital exclusion	Strong relationships between GPs and community groups, support from local authorities	Effective in addressing social determinants of health, but more funding and structured evaluation needed for long-term success [28]
Tull [28]	Rapid desk-based review of research evidence, systematic reviews, interviews, grey literature	Effectiveness of strategies to reduce vaccine hesitancy, guidance for developing vaccine policies	No formal evaluation frameworks specified	Effective strategies include communication-based training for healthcare workers, use of religious/community leaders, and tailored mass media campaigns	Strategies aimed at marginalised populations effectively addressed inequalities and improved vaccine uptake	Improved vaccine uptake in settings with previously low immunisation rates	Not assessed, but improvements in uptake expected to ease burden on healthcare systems	Funding constraints, misinformation, mistrust of vaccines and healthcare providers	Religious leaders, tailored communication strategies, mass media, and collaboration between stakeholders	Addressing vaccine hesitancy requires a tailored approach with active involvement of community influencers; further evaluation of long-term impacts needed
Kassianos et al. [29]	Qualitative analysis of interviews with healthcare professionals and managers from North West London	Improved collaboration, team integration, and holistic care outcomes	No formal framework; outcomes derived from qualitative themes	Improved coordination between primary and secondary care, enhanced multidisciplinary collaboration and learning opportunities	Positive impact by reducing fragmentation in patient care, especially for elderly and diabetic populations	Improvements in holistic and coordinated care delivery, especially in chronic disease management	Not directly assessed, but improved care coordination expected to reduce unnecessary admissions and enhance resource allocation	Challenges included time constraints for practitioners and lack of standardised evaluation tools	Facilitated through structured multidisciplinary team meetings and shared goals among healthcare providers	Integrated care initiatives improve coordination and outcomes; formal evaluations needed for resource impact assessment
Change Starts With Me Evaluation Team [30]	Mixed methods evaluation (focus groups, workshops, reflective reviews)	Improved leadership skills, team effectiveness, and patient outcomes	No formal framework; reflective reviews and co-created frameworks used for analysis	Participants gained confidence and skills in leadership, team engagement, and quality improvement	Addressed health inequalities through PCN-led community initiatives and projects addressing local needs	Improved team morale, patient care, and service quality across participating PCNs	Not directly assessed, but improved team functionality and service delivery expected to optimise resource use	Barriers included resistance to change, time constraints for training, and limited initial buy-in from some participants	Strong focus on peer consulting, multidisciplinary collaboration, and leadership skill development	Multi-professional leadership programs enhance team capabilities, community engagement, and health outcomes; scalable for broader implementation
Public Health England [31]	Mixed-methods evaluation including interviews, focus groups, surveys, and document analysis	Increased stakeholder engagement, systems thinking adoption, action plan creation	No formal evaluation framework specified	Positive impacts on systems thinking, collaboration, and shifting focus toward broader determinants of health	Highlighted inequality reduction through engagement of multiple sectors addressing obesity-related health disparities	Improved understanding of obesity causes, increased collaboration; early signs of change in approach but too early to assess full impact	Not directly assessed, but increased engagement and system-wide thinking expected to improve resource utilisation	Funding constraints, lack of senior leadership buy-in, difficulty securing cross-sector engagement	Strong local leadership, well-connected teams, multi-sectoral collaboration, and sustained stakeholder engagement were key facilitators	Whole systems approaches can bring about systems-level change, but long-term commitment, resources, and multi-sector collaboration are needed for success
Future Spaces Foundation [32]	Qualitative insights gathered through expert panels and case study analysis	Improved sense of community, increased social interaction, enhanced access to public spaces	No formal frameworks used, but design concepts and proposals evaluated qualitatively	Urban design can exacerbate or reduce loneliness; accessible, inclusive public spaces and housing designs help foster social connections	Highlighted how built environments can help reduce loneliness, particularly among vulnerable populations such as the elderly and migrants	Improved mental well-being through increased social engagement and access to public spaces	Not directly assessed, but improved urban design expected to reduce demand for mental health and social care services	Challenges in securing funding for large-scale urban redesign projects; cultural barriers to adopting new housing and public space models	Collaboration between urban planners, local authorities, and communities; innovative design thinking that centres on community needs	Proposes that urban design plays a critical role in reducing loneliness and fostering social cohesion; calls for policy changes and inclusive urban planning approaches
Cheetham et al. [33]	Realist evaluation, combining routine data analysis with qualitative interviews and focus groups	Improved mental health, physical well-being, social interaction, reduced social isolation	No formal evaluation framework specified; routine monitoring data supplemented with qualitative insights	Integrated wellness services improve mental well-being, promote social interaction, and reduce social isolation, particularly when addressing multiple health and social concerns simultaneously	Positive impact on addressing inequalities, particularly in deprived and disadvantaged communities	Improved mental health, well-being, and physical activity, with significant self-reported improvements in confidence and self-efficacy	Not directly assessed, but expected to reduce long-term pressure on health and social care services	Limited funding and sustainability, challenges in capturing long-term outcomes in routine data	Strong leadership, flexible service delivery, strong relationships between staff and participants	Integrated wellness services addressing complex health and social issues can significantly improve mental and physical health, but require long-term investment and flexible, community-driven approaches
Reynolds [34]	Observational and interview-based ethnographic analysis	Improved resident engagement, negotiation of boundaries, connection of community members	No formal evaluation framework	Community engagement led to positive local impacts, but boundary work highlighted ongoing inclusion and exclusion dynamics	Addressed social inequalities through resident empowerment, but risks of entrenching existing inequalities	Improved engagement, but impacts on health outcomes were not directly assessed	Not directly assessed, but the model aimed to reduce reliance on external agencies and improve local resource management	Funding constraints, tensions between community interests, and challenges in representing diverse interests	Resident-led decision-making, support from Local Trust, and strong engagement with community representatives	Community empowerment and engagement can positively impact local governance and decision-making, but boundaries must be navigated to avoid exclusion
Orton et al. [35]	Ethnographic fieldwork and systems analysis	Enhanced resident engagement, new community networks, and increased sense of ownership	No formal evaluation framework specified	Empowerment and resident-led decision-making were key to the initiative’s success in improving social outcomes and community cohesion	Positive impact on addressing social inequalities through resident-led projects and engagement	Improved social cohesion, local decision-making, and community empowerment	Not directly assessed, but improved resource management was noted in several areas	Funding constraints, conflicts between sub-areas, and difficulty engaging some marginalised groups	Strong resident leadership, flexible initiative design, and integration with local authorities and external partners	Resident-led, area-based empowerment initiatives can drive positive social change, but require long-term support, funding, and conflict resolution mechanisms
Findlay et al. [36]	Evaluation based on community participation, empowerment, and local health outcomes	Improved community capacity, social engagement, and reduced health inequalities	No formal evaluation framework but iterative feedback incorporated through qualitative data collection	Positive impact on community well-being, health, and social inclusion through asset-based approaches	Significant positive impact on reducing health inequalities, particularly in the most disadvantaged areas	Improved mental health, social cohesion, and participation rates	Not directly assessed but expected improvements through integrated resource use and community-driven support	Funding challenges, complexity in scaling up the approach, and ensuring fidelity of the model across locations	Strong leadership from local authorities and community stakeholders, effective use of local assets and volunteers	Well Communities successfully integrated into mainstream public health efforts, but long-term sustainability requires continued investment and adaptation
Ejbye et al. [37]	Qualitative evaluation of learning and practical implementation across the five sites	Improvements in well-being, confidence, mental health, and self-management	No formal evaluation framework; qualitative insights and practical lessons from partner sites	Positive improvements in self-management, well-being, and patient activation; peer support was found effective in improving mental health outcomes	Addressed social isolation, improved access to care, and empowered marginalised groups to engage in health services	Improved mental health, self-management, and quality of life for patients	Not directly assessed, but enhanced service delivery through community engagement and self-management	Lack of sustainable funding and long-term commitment, digital exclusion, and limited scalability	Strong community engagement, flexible service delivery, and peer-led interventions were key facilitators	Person- and community-centred approaches improve health and well-being, but sustainability and scalability require further investment and policy support
Groene et al. [38]	Quasi-experimental study and biannual surveys, supported by routine data analysis	Improved population health outcomes, satisfaction, and cost savings	No formal evaluation framework specified	Positive health outcomes and increased life expectancy; 45.4% of patients reported healthier lifestyles	Positive impact on health inequalities through integrated care services and community engagement	Improved life expectancy (1.4 years) and better health-related quality of life for patients	€5.5 million annual savings in 2013 through reduced health service overuse, improved prescription practices	Funding and stakeholder collaboration challenges during early implementation, long-term sustainability risks	Strong local leadership, patient engagement, financial accountability, and use of big data for monitoring	Integrated care models like Gesundes Kinzigtal can lead to significant improvements in population health, but require strong governance, patient engagement, and sustainable funding
Farmer et al. [39]	Workshops and thematic analysis of community inputs	Identified local health priorities, such as emergency triage, health volunteering, and wellbeing improvement	No formal evaluation framework specified; findings based on participatory action methods	New service models were designed in some communities, demonstrating that participation impacts service design in rural settings	Not directly assessed, but the approach aimed to reduce inequalities in access to healthcare by empowering communities	Improved engagement in rural primary care services and increased sense of ownership over healthcare provision	Not directly assessed, but local service design intended to optimise resource use within existing budgets	Lack of community engagement in some areas, local resistance to participation, logistical challenges in workshop attendance	Strong social capital and willingness to engage among some communities, local knowledge of healthcare needs, and flexible service design	Community participation can lead to service models that address local needs, but community receptiveness varies. Tailored approaches are needed to engage all communities
Ben Collins, The King’s Fund [40]	Qualitative evaluation of system redesign, focus on leadership structure and community engagement	Improved life expectancy, lower hospital admissions, reduced A&E use, improved satisfaction	No formal evaluation framework specified but aligned with performance indicators (e.g., Baldrige Excellence Framework)	Positive impact on addressing health inequalities by empowering Alaska Native people to co-design their care systems	Improved life expectancy, health outcomes, patient satisfaction, and care delivery efficiency	Reduction in hospital admissions, A&E visits, and specialist referrals; cost savings achieved	Not directly assessed	Funding challenges during implementation, resistance to shifting to customer-owner-driven model	Strong community leadership, robust training for staff, integration of traditional healing with modern healthcare approaches	Community-driven models of care can dramatically improve health outcomes and resource efficiency when community ownership and leadership are at the core
NIHR [41]	Qualitative analysis based on pilot implementation and case studies	Increased engagement in health screenings, reduction in GP appointments	No formal evaluation framework specified	CHWWs improved community trust and health screening rates, reducing GP workload and identifying previously unknown high-risk patients	Positive impact by reaching underserved populations and reducing health inequalities	Improved access to preventive care, mental health services, and chronic disease management	Reduced GP workload through proactive outreach, lower emergency care utilisation	Funding and workforce capacity challenges, cultural adaptation of the CHWW model to different regions	Strong community engagement, cultural competence, and integration with local health services	CHWWs have shown early positive impacts on community health and service use, with potential for national scalability with sustainable funding
NHS Confederation [42]	Qualitative and quantitative assessment, focusing on social outcomes and reduction in GP visits	Reduced non-clinical GP visits by one-third, social return on investment of £4 for every £1 invested	No formal framework, but mixed qualitative and quantitative methods used	Significant reduction in non-clinical GP visits, improved social outcomes, increased community participation	Positive impact by reducing health inequalities related to social isolation and loneliness	Improved mental well-being, reduced social isolation, increased community involvement	Reduced non-clinical GP visits by one-third, improving service efficiency	Initial slow uptake of referrals from GPs, difficulty in embedding asset-based approaches	Strong local leadership, effective cross-sector collaboration, LACs embedded in communities	LACs successfully reduced loneliness and improved health outcomes; asset-based models are effective for tackling social issues
Dale McMurchy Consulting [43]	Analysis of emergency department (ED) utilisation data compared to expected utilisation for CHC clients	CHC clients showed a 21% lower-than-expected rate of ED visits, with estimated savings of $27 million annually	No formal evaluation framework but based on observed vs. expected ED utilisation rates	CHCs reduced ED utilisation rates, showing greater cost-effectiveness compared to other primary care models in Ontario	Significant impact in reducing health inequalities by addressing social determinants of health	Improved access to care, better health management, and reduced avoidable hospitalizations	Reduced ED visits, saving $27 million annually due to fewer hospitalizations and emergency visits	Funding constraints and lack of clear evidence on the cost-effectiveness of programs	Strong integration between health and social services, comprehensive care model tailored to equity-seeking populations	CHCs effectively reduce healthcare utilisation and improve patient outcomes, though more evidence on cost-effectiveness is needed
NHS England [44]	Qualitative and quantitative assessment of service access and mental health outcomes	Reduced waiting times for mental health services, improved educational and employment outcomes	No formal evaluation framework specified	100 young people accessed services, 20 re-entered education, and 20 into employment in the first 10 months	Addressed mental health inequalities in children and young people from deprived areas	Improved access to mental health services and educational/employment support for young people	Not directly assessed, but improved service access expected to reduce healthcare utilisation	Challenges in embedding new models of care and long-term funding uncertainties	Strong community engagement and collaboration between health, social care, and voluntary sectors	INT successfully reduced mental health service waiting times and improved education/employment outcomes for young people
Institute of Health Equity [45]	Quantitative analysis of life expectancy, mortality rates, and social determinants of health	Life expectancy stagnation, increased mortality in deprived areas, widening health inequalities	No formal evaluation framework specified	Health inequalities have worsened, especially in deprived areas, with life expectancy declining for women in deprived areas	Worsening health inequalities, particularly among ethnic minorities and disadvantaged communities	Decline in life expectancy in the most deprived areas, increased years spent in ill health	Not directly assessed, but potential long-term impacts due to increased pressure on health and social services	Funding cuts, austerity measures, worsening social determinants of health	Effective community action and local government-led initiatives (e.g., Marmot cities)	Health inequalities are not inevitable and can be significantly reduced with social justice approaches

### Characterisation of IN models

Integrated Neighbourhoods (INs) were conceptualised in various ways to meet specific community health needs through localised, cross-sector collaboration. Common across the models was an emphasis on integrating health and social care with other sectors, such as, public health, community organisations, and the creative industries, to address health inequalities and promote well-being at the hyper-local level (Tables 1 and 2) [17].

Some models focussed on community empowerment and local leadership. For instance, one model supported community-driven health initiatives that emphasised preventive care and tackled social determinants in underserved areas [19]. Other models, like ‘Arts on Prescription,’ emphasise non-clinical, creative approaches to mental well-being, illustrating the diversity in IN methodologies [18].

INs also varied in structure and evaluation methods. While some relied on participatory feedback (e.g., focus groups) [21], others incorporate more structured metrics, such as social and mental health outcomes assessed through validated scales.

Direct community-based participatory involvement in service design and delivery was noted across INs. These models used workshops, focus groups, and co-production activities to tailor services to local needs and enhance community ownership [18, 19, 21]. Cross-sector partnership approaches allowed for collaboration between healthcare, social care, and voluntary sectors, creating holistic care provision in INs. The Bradford Inequalities Research Unit, for example, partnered with NHS England, local councils, and academic institutions to integrate support pathways and deliver targeted interventions [21]. Partnerships in models further enabled workforce integration and enhanced resource utilisation [41]. In addition, Asset-Based Community Development (ABCD) methodologies leveraged local strengths and resources. For example, local artists contributed to mental health and social inclusion through creative programs and community assets were mobilised to address social determinants of health [17, 36].

INs prioritised prevention-focused programs aimed at addressing broader social determinants of health, including housing, employment, and mental health, which were integral to many IN initiatives. [19, 31] Moreover, following on from the pandemic, hybrid approaches of service delivery were deployed, such as blending digital and in-person services. This aimed to maintain accessibility during service disruption [20]. Lastly, some INs incorporated peer support and community champion models, which foster health engagement through local advocates [37].

### Evaluation and impact assessment of IN models

Across the studies, evaluation methodologies range from qualitative feedback to structured quantitative assessments, often focusing on health outcomes, social inclusion, and resource utilisation (Table 1). However, many studies relied upon informal feedback without standardised frameworks.

Qualitative and mixed-methods approaches, gathering insights through participant feedback, focus groups, and case studies were frequently used to assess service relevance, trust-building, and community engagement [18, 41]. Some INs employed structured quantitative tools to measure outcomes related to mental health, social inclusion, and service utilisation to quantify improvements in mental well-being, as well as health data to track engagement in obesity prevention activities and systems-level change [17, 31].

Two studies incorporated data-driven or comparative analyses to assess program effectiveness, targeting health inequalities and tracking changes in healthcare usage. The Bradford Inequalities Research Unit applies analytics to measure reductions in hospital admissions and emergency visits [21]. Similarly, in Ontario, emergency department utilisation rates for community health centre clients against expected averages, highlighting resource savings and cost-effectiveness [43]. Other models evaluated economic and social returns to assess the financial impact of reduced healthcare usage. For example, in York, a social return of £4 for every £1 invested was calculated, due to reductions in non-clinical general practitioner visits and increased community engagement [42].

### Barriers and facilitators

A detailed breakdown of barriers and facilitators can be seen in Table 2. Common reported barriers included funding constraints and sustained funding, digital exclusion, workforce limitations, and organisational resistance to change. Factors facilitating the success and sustainability of IN models included strong community engagement, integration of a strong motivated workforce, and positive leadership and commitment from local authorities.

### Key domains of IN and proposed framework

**Fig. 2:**
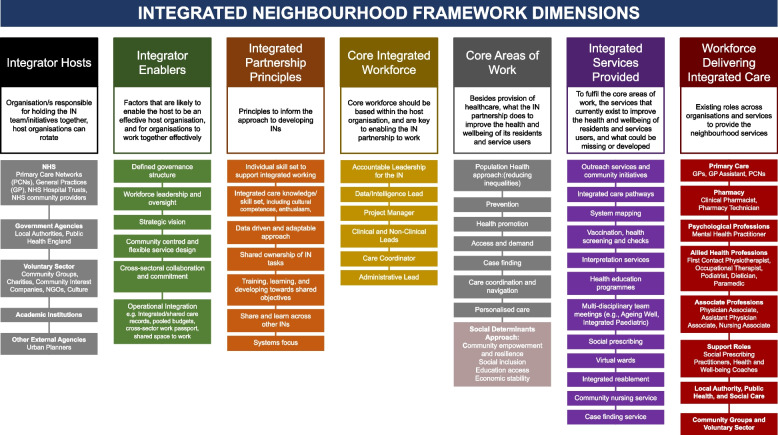
Integrated neighbourhood framework domains

Overall, seven key domains were identified from the included studies, the integrator host; integrator enablers; integrator partnership principles; core integrated workforce; core areas of work; integrated services provided; and type of workforce delivering integrated care (Table 3 and Fig. 2).

**Table 3:** Mapping of studies to identified key integrated framework domains

Study	Integrator Host	Integrator Enablers	Integrated Partnership	Core Integrator Workforce	Core Areas of Work	Services Provided	Workforce in Place
Bungay et al. [17]	Local community organisations and voluntary sectors	Enthusiasm from GPs, local voluntary sectors, community-led initiatives	Collaboration between health services, community artists, and local organisations	Community practitioners, GPs, voluntary sector workers	Mental well-being, social inclusion	Creative arts interventions, social inclusion programs	Local artists, GPs, community practitioners, voluntary sector staff
Edmonton Community Partnership [18]	Edmonton Community Partnership	NHS-voluntary sector-community collaboration	Cross-sector collaboration between NHS, local authorities, and voluntary sectors	Community organisers, healthcare professionals, voluntary sector leaders	Reducing health inequalities, improving trust in services	Workshops, focus groups, community-driven activities	Community leaders, local healthcare professionals, voluntary sector members
Lent et al. [19]	NHS, local authorities, community groups	National support, community leadership, prevention-focused commitment	Partnership across NHS, local authorities, and community stakeholders	Healthcare professionals, community leaders, local council staff	Prevention-based care, community empowerment	Preventive health services, community empowerment activities	Healthcare workers, community representatives, local authority staff
Health and Social Care Alliance Scotland (the ALLIANCE) [20]	Local authorities and HSCPs	Emergency legislation, flexible service models, digital transformation	Collaboration between local authorities, HSCPs, and third sector organisations	Local authority and HSCP staff, third sector partners	Digital inclusion, flexible support models during COVID- 19	Virtual services, hybrid service models combining remote and in-person care	Local authority and HSCP staff, third sector staff
South et al. [23] and Curry et al. [46]	Community members, practitioners, local authorities	Community ownership, long-term commitment, organisational buy-in	Collaboration between community members, practitioners, and local authorities	Local community members, public health professionals	Community engagement, empowerment, asset-based approaches	Participatory methods, asset mapping, community-led interventions	Community members, local practitioners, public health professionals
Dickerson et al. [21]	NHS England, local authorities, academic institutions	Targeted interventions, use of big data (Connected Bradford)	Collaboration between NHS, local authorities, and academic teams	Healthcare professionals, academic researchers	Health inequalities, premature mortality	Integrated care pathways, community readiness for interventions	Local healthcare providers, academic teams, welfare advisors
Such et al. [22]	General practices, NHS, and external agencies	Commitment to equitable care, tailored outreach efforts	Collaboration with local charities, community groups, and police	General practitioners, community health workers	Culturally competent care, trauma-informed services	Screening, vaccination, interpreter services, health checks	GPs, interpreter services, community support workers
South et al. [23] and Curry et al. [46]	NW London Health system led to primary, secondary, community, Mental health and VSO	Agreed governance and funding model, strategic vision	Data driven agreed outcomes, clear tasks for teams, high engagement,	Multidisciplinary teams	Wellbeing, social capital, community infrastructure	Co-production, participatory learning, community-driven action	Community members, local government staff, volunteers
LGA [24]	Local government and public health leadership	Long-term partnerships, community-centred approach	Networks, relationships, mutual support	Public health teams, local government officials	Asset-based approaches, mutual aid	Community empowerment, social capital, support networks	Local government staff, community organisations, volunteers
Merron Simpson, Royal College of General Practitioners, Health Creation Alliance [25]	PCNs, local partners	Collaboration tools, community engagement strategies	Partnerships between primary care, local authorities, and voluntary sectors	GPs, local authority representatives, voluntary sector workers	Reducing health inequalities, improving mental health	Community-led initiatives, mental health support, strategic partnerships	GPs, local council staff, community sector partners
Public Health Wales [26]	NHS, local organisations	Flexible support, personalised approach	Collaboration between social prescribing practitioners, local authorities, and community organisations	Social prescribing practitioners, link workers	Loneliness, mental health, caregiver support	Personalised support conversations, connections to local groups	Social prescribing practitioners, local community groups, NHS staff
Tull [28]	Government stakeholders, healthcare providers, NGOs	Community engagement strategies, tailored communication	Collaboration between healthcare providers and policy makers	Healthcare professionals, community influencers	Building vaccine confidence, addressing health misinformation	Tailored communication strategies, community outreach initiatives	Healthcare workers, community leaders, NGOs
Public Health England [31]	Local councils, Public Health England (PHE)	Stakeholder engagement, multi-sector collaboration	Collaboration between local authorities, public health teams, and community partners	Local government staff, public health teams	Obesity prevention, systems thinking	System mapping, workshops, action planning	Public health professionals, local council staff, community groups
Future Spaces Foundation [32]	Future Spaces Foundation	Collaboration between urban planners and communities	Multidisciplinary collaboration with architects, social scientists, and policymakers	Urban designers, social scientists, policymakers	Urban design, social connections, reducing loneliness	Designing inclusive public spaces and housing	Urban designers, local government staff, social scientists
Cheetham et al. [33]	NHS, local authority	Strong leadership, flexible service delivery	Partnership between local authority, NHS, and community capacity-building staff	Wellness coaches, community workers	Mental well-being, social interaction, reducing social isolation	Group sessions, tailored 1:1 support	Wellness coaches, NHS staff, community workers
Reynolds [34]	Local residents, supported by Local Trust	Resident-led leadership, community decision-making	Partnership between residents, local authorities, community workers	Local residents, community workers	Community engagement, resident-led empowerment	Consultation, resident participation, decision-making committees	Local residents, community representatives, local authority staff
Orton et al. [35]	Local residents, Local Trust	Resident leadership, flexible initiative design	Partnership between residents, local authorities, and external partners	Local residents, council workers, external partners	Community empowerment, collective action	Workshops, events, decision-making meetings	Community members, local council staff, external partners
Findlay et al. [36]	Local authorities, community members, public health teams	Long-term partnerships, community assets	Partnership between public, private, and third sectors	Local authority staff, public health teams, community members	Community well-being, resilience, empowerment	Community action plans, volunteering initiatives	Public health teams, local authority staff, community members
Ejbye et al. [37]	NHS, community organisations, voluntary sectors	Community engagement, peer support	Cross-sector collaboration between community organisations, NHS, and voluntary sectors	Community health workers, NHS staff	Peer support, self-management, health coaching	Group activities, workshops, community support	Community health workers, local council staff, NHS staff
Groene et al. [38]	Regional integrator, sickness funds	Patient engagement, use of big data	Collaboration between healthcare providers and sickness funds	Healthcare professionals, patient advisors	Population health management, integrated care	Shared decision-making, health education programs	Healthcare providers, patient advisors, community stakeholders
Farmer et al. [39]	NHS Highland, community groups	Strong social capital, local knowledge	Collaboration between local practitioners and community members	Healthcare providers, community members	Primary healthcare, emergency care, wellbeing improvement	Workshops, planning exercises, local service design	Community members, local healthcare providers, NHS staff
Ben Collins, The King’s Fund [40]	Alaska Native people (customer-owners)	Customer-owner leadership, integration of traditional healing	Multidisciplinary collaboration between health and social services	Primary care providers, traditional healers, community leaders	Holistic wellness, relationship-based care	Primary care, mental health, dental services, traditional healing	Primary care providers, traditional healers, community leaders
NIHR [41]	Westminster community services, CHWWs	Cultural competence, integration with local services	Collaboration between GPs, CHWWs, and local authorities	CHWWs, GPs, community services staff	Health promotion, chronic disease management, social care navigation	Home visits, workshops, tailored support	CHWWs, GPs, community services staff
NHS Confederation [42]	City of York Council, health and voluntary sectors	Local Area Coordination (LAC), embedded in communities	Partnership between LAC teams, GPs, and local groups	Local Area Coordinators, GPs, community groups	Reducing loneliness, social isolation	Walking groups, community volunteering, LAC initiatives	Local Area Coordinators, GPs, community group leaders
Dale McMurchy Consulting [43]	Community Health Centres (CHCs)	Comprehensive care model, focus on equity	Collaboration between CHCs, community organisations, and public health sectors	Interprofessional teams, community health workers	Primary care, social care, health promotion	Comprehensive primary healthcare, social services, health promotion activities that address social determinants of health	Interprofessional teams composed of social workers, community health workers, physicians, and public health professionals
Kassianos et al. [29]	NHS and primary care practices in North West London	Structured multidisciplinary meetings, leadership support, shared learning	Partnership between NHS trusts, primary care, and community health services	Healthcare professionals, including GPs, geriatric specialists, and social workers	Integrated care, chronic disease management	Multidisciplinary team meetings, care planning, coordination of services	Primary care teams, community health professionals, geriatric specialists
Change Starts With Me Evaluation Team [30]	Medway Clinical Commissioning Group, North Kent Training Hub	Multi-professional leadership program, peer consulting, and community engagement	Partnership across PCNs, clinical commissioning groups, and local authorities	PCN teams, including GPs, practice nurses, practice managers, and pharmacists	Leadership development, community health improvement	Leadership masterclasses, co-created service improvement projects, community-focused initiatives	PCN teams, multidisciplinary healthcare professionals

#### Integrator hosts

The role of integrator hosts in enabling cohesive, community-focused healthcare initiatives is pivotal in driving system-wide improvements. Across the studies, integrator hosts range from local authorities and voluntary sectors to national health systems, serving as anchor institutions in diverse social and geographical contexts (Table 3).

Notable examples include the Edmonton Community Partnership (a collaboration of schools), which fostered robust collaboration between the NHS, local authorities, and voluntary sectors to address health inequalities and rebuild trust in public services. This integrator host enabled cross-sector workshops and community-driven activities, leveraging local leadership to amplify its impact [18].

The Medway & Swale Multi-Professional Leadership Programme, a PCN led partnership with the Clinical Commissioning Group (CCG) in the UK, highlights the transformative potential of multidisciplinary leadership initiatives. This program employed peer consulting and leadership masterclasses, culminating in co-created service improvement projects. These interventions not only enhanced team capabilities but also fostered community engagement through tailored projects such as social prescribing initiatives [30].

Collectively, these integrator hosts illustrate the diverse strategies and configurations necessary to navigate complex healthcare ecosystems.

#### Integrator enablers

The integration of health and social care systems hinges on a robust set of enablers that facilitate collaboration and sustainability across sectors including patients who are served. Key enablers identified in the literature include leadership, clear shared vision with governance structures, practical considerations such as places to meet and ability to share records and data. (Table 3).

The North West London health system brought multiple partners across a large population and led virtual networks to conduct structured multidisciplinary collaboration. Regular team meetings across primary and secondary care providers facilitated care planning and coordination for chronic disease management. This way of working was enabled through a clear shared strategic vision, established governance and clear funding for partners [29].

Leadership was a core focus of programs like the Medway & Swale Multi-Professional Leadership Programme, which employed peer consulting and co-created service improvement projects to empower multidisciplinary teams in addressing health inequalities and improving community health outcomes [30].

The adaptability of service delivery models was particularly evident during the COVID- 19 pandemic. The Learning from Changes to Social Care initiative highlighted the importance of flexible digital transformation to maintain access to care. Hybrid service models combining virtual and in-person support mitigated the challenges of digital exclusion while ensuring continuity of services [20].

These examples illustrate the multifaceted enablers which contribute to the success of integrative health initiatives. By aligning cultural, community, and systemic resources, these programs demonstrate the potential to enhance equity, trust, and resilience in health systems.

#### Integrator partnership principles

INs depend on robust partnerships that unite health systems, local authorities, voluntary sectors, and community organisations (Table 3). These partnerships enable multidisciplinary approaches that leverage expertise across public health, social care, and community empowerment to address complex health challenges.

An initiative, hosted by Public Health England and local councils, utilised system mapping and stakeholder engagement to tackle obesity at the population level. Through collaborative system mapping, local stakeholders identified interconnected causes of obesity, such as food access, physical activity environments, and socioeconomic factors. Stakeholder engagement brought together public health teams, local authorities, schools, and community groups to align actions and build strong social networks. Efforts also included action planning to address both systemic and individual-level interventions, shifting the focus from lifestyle changes to structural determinants of health, like urban planning and access to nutritious food. Capacity-building workshops and training enhanced stakeholders’ understanding of systems science, while dynamic feedback loops allowed for iterative adaptation and continuous improvement [31].

Similarly, collaborations between the NHS, local authorities, and community organisations have promoted co-production and participatory learning to enhance social capital and community infrastructure. These efforts underline the importance of fostering resilient communities through shared ownership and collective action [23].

The Adapting Primary Care for New Migrants program provides a comprehensive model addressing health inequalities for underserved populations through a robust network of partnerships. These connections were established through strategic coordination between general practitioners, community health workers, and local charities. Efforts included collaborative initiatives like signposting patients to welfare and housing support, coordinating with schools, and integrating services such as social care and mental health support. The program also leveraged tailored outreach activities, drop-in clinics, and extended appointment times to better serve migrant populations. This approach fostered culturally competent and trauma-informed care while addressing wider social determinants of health, demonstrating how partnerships and adaptations can drive equity-oriented healthcare provision [22].

#### Core integrator workforce

IN models rely on a multidisciplinary workforce that integrates traditional healthcare professionals with community leaders, project managers, and other stakeholders to drive strategic goals and monitor progress effectively (Table 3). These teams allow for services to be tailored to local needs and aligned with measurable outcomes.

The Community-Powered Edmonton Initiative demonstrated leadership through collaborative coordination by community organisers, healthcare professionals, and voluntary sector leaders. The initiative was guided by project managers and coordinators who ensured alignment between the NHS, local authorities, and community groups. Regular workshops and focus groups provided feedback loops to assess progress on goals like improving trust in services and reducing health inequalities [18].

In addition, the Live Well Gateshead Initiative combined the efforts of wellness coaches, NHS staff, and community workers, with strong leadership from local authorities. Project coordinators ensured tailored interventions were delivered through one-on-one support and group sessions. Progress was monitored using community feedback and metrics such as reductions in social isolation and improvements in mental well-being [33].

Lastly, one initiative illustrated the integration of local government staff, public health professionals, and volunteers under the leadership of coordinators and project managers. These leaders oversaw participatory learning activities and co-production efforts, evaluating outcomes through community-driven action plans and structured reporting systems. Metrics of success included increases in social capital, community engagement, and infrastructure development [23].

#### Core areas of work

The core areas of work within IN are tailored to the unique needs of their local populations, addressing a spectrum of population health and social priorities such as prevention, well-being, health promotion, and social inclusion (Table 3). By targeting social determinants like housing, employment, and education, these models aim to foster sustainable health improvements.

The Public Health England approach for tackling obesity illustrates a prevention-focused model, working with local councils. Through system mapping, workshops, and stakeholder engagement, this initiative emphasized cross-sector collaboration to address obesity at the population level. The working model used systems thinking and action planning to build community-wide strategies for health improvement [31].

The Adapting Primary Care for New Migrants program showcases a focus on equitable access to culturally competent care. This initiative emphasized screening, vaccination, and interpreter services, while addressing trauma-informed care needs to bridge gaps for underserved populations. The program’s tailored outreach highlighted the importance of community engagement in delivering responsive care [22]. Evidently, there is diversity of IN priorities, demonstrating that core areas of work can be responsive to immediate community needs.

The IN work programmes centre on a population health approach, social determinants of health and patient centred care. The characterization of the work is not clear cut and there is clearly overlap. An IN benefits from this focus to support its resource allocation as well as evaluation and sustainability planning.

#### Services provided from IN working

INs combine traditional health services with community-driven support systems (Table 3). For example, ‘Social Prescribing’ [26, 40]. Service delivery is often tailored to community priorities, as seen in rural healthcare design projects in Scotland, which integrate local knowledge and emergency care into service planning [39]. This flexibility enables INs to adapt services dynamically to meet evolving health needs.

#### Workforce delivering the IN care

INs utilise a diverse, multidisciplinary workforce tailored to local needs, combining clinical, community, and specialised roles. This includes healthcare providers, community practitioners, social workers, and unique roles like Local Area Coordinators and wellness coaches (Table 3). The flexible workforce structure allows INs to deliver comprehensive, community-centred care and effectively address complex health and social issues, reinforcing the overall impact on health equity and well-being [17, 20, 42].

#### Risk of bias assessment

A detailed risk of bias assessment for the included studies is presented in Table 4. Several studies employed qualitative methodologies, such as interviews, focus groups, and case studies, which are inherently prone to issues of reflexivity and researcher influence. The absence of explicit discussions about researcher bias and lack of standardised data collection protocols were recurrent limitations across these studies [17, 23, 38]. While qualitative insights provided valuable narratives, they often lacked rigorous integration with quantitative findings, which limited their explanatory power.

**Table 4:** Risk of bias using the mixed-methods appraisal tool for included studies

Study	1.1	1.2	1.3	1.4	1.5	2.1	2.2	2.3	2.4	2.5	3.1	3.2	3.3	3.4	3.5	5.1	5.2	5.3	5.4	5.5
Bungay et al. [17]	1	1	0.5	1	1	0	1	1	0	0	1	1	0	1	1	0.5	0.5	0.5	0.5	0.5
Edmonton Community Partnership [18]	1	1	0.5	0.5	1	0	1	1	0	0	1	1	0	1	1	0.5	0.5	0.5	0.5	0.5
Lent et al. [19]	1	1	0.5	0.5	1	0	1	1	0	0	1	1	0	1	1	1	0.5	0.5	0.5	0.5
Health and Social Care Alliance Scotland (the ALLIANCE) [20]	1	0.5	0.5	0.5	1	0	1	1	0	0	1	1	0	1	1	0.5	0.5	0.5	0.5	0.5
Dickerson et al. [21]	1	1	1	1	1	0	1	1	0	0	1	1	0	1	1	1	1	1	1	1
Such et al. [22]	1	0.5	0.5	0.5	0.5	0	1	1	0	0	1	1	0	1	1	1	1	0.5	0.5	0.5
South et al. [23]	1	1	1	1	1	0	1	1	0	0	1	1	0	1	1	1	1	1	1	1
LGA [24]	1	1	0.5	0.5	0.5	0	1	1	0	0	1	1	0	1	1	0.5	0.5	0.5	0.5	0.5
Merron Simpson, Royal College of General Practitioners, Health Creation Alliance [25]	1	1	1	1	1	0	1	1	0	0	1	1	0	1	1	1	0.5	0.5	0.5	0.5
East Midlands Academic Health Science Network [27]	1	0.5	0.5	0.5	1	0	1	1	0	0	1	1	0	1	1	1	1	1	1	1
Change Starts With Me Evaluation Team [30]	1	1	1	1	1	0	1	1	0	0	1	1	0	1	1	1	1	1	1	1
Public Health England [31]	1	1	1	1	1	0	1	1	0	0	1	1	0	1	1	1	0.5	0.5	0.5	0.5
Future Spaces Foundation [32]	1	0.5	0.5	0.5	0.5	0	1	1	0	0	1	1	0	1	1	0.5	0.5	0.5	0.5	0.5
Cheetham et al. [33]	1	0.5	1	0.5	0.5	0	1	1	0	0	1	1	0	1	1	1	1	1	1	1
Reynolds [34]	1	1	1	1	1	0	1	1	0	0	1	1	0	1	1	1	1	1	1	1
Orton et al. [35]	1	1	1	1	1	0	1	1	0	0	1	1	0	1	1	1	1	1	1	1
Findlay et al. [36]	1	1	0.5	0.5	1	0	1	1	0	0	1	1	0	1	1	1	0.5	0.5	0.5	0.5
Ejbye et al. [37]	1	0.5	0.5	1	0.5	0	1	1	0	0	1	1	0	1	1	1	1	1	1	1
Groene et al. [38]	1	1	1	1	1	0	1	1	0	0	1	1	0	1	1	1	1	1	1	1
Farmer et al. [39]	1	1	1	0.5	0.5	0	1	1	0	0	1	1	0	1	1	1	1	0.5	0.5	0.5
The King’s Fund [40]	1	1	0.5	1	1	0	1	1	0	0	1	1	0	1	1	1	1	1	1	1
NIHR [41]	1	1	1	1	1	0	1	1	0	0	1	1	0	1	1	1	1	1	1	1
NHS Confederation [42]	1	1	1	1	1	0	1	1	0	0	1	1	0	1	1	1	1	1	1	1
Dale McMurchy Consulting [43]	1	0.5	1	1	1	0	1	1	0	0	1	1	0	1	1	1	1	1	1	1
NHS England [44]	1	1	1	1	1	0	1	1	0	0	1	1	0	1	1	1	1	1	1	1
Institute of Health Equity [45]	1	1	0.5	0.5	1	0	1	1	0	0	1	1	0	1	1	1	1	1	1	1

1.1, Is the qualitative approach appropriate to answer the research question? 1.2, Are the qualitative data collection methods adequate to address the research question? 1.3, Are the findings adequately derived from the data? 1.4, Is the interpretation of results sufficiently substantiated by data? 1.5, Is there coherence between qualitative data sources, collection, analysis, and interpretation? 2.1, Is randomization appropriately performed? 2.2, Are the groups comparable at baseline? 2.3. Are there complete outcome data? 2.4, Are outcome assessors blinded to the intervention provided? 2.5, Did the participants adhere to the assigned intervention? 3.1, Are the participants representative of the target population? 3.2, Are measurements appropriate regarding both the outcome and intervention (or exposure)? 3.3, Are there complete outcome data? 3.4, Are the confounders accounted for in the design and analysis? 3.5, During the study period, is the intervention administered (or exposure occurred) as intended? 5.1, Is there an adequate rationale for using a mixed methods design to address the research question? 5.2, Are the different components of the study effectively integrated to answer the research question? 5.3, Are the outputs of the integration of qualitative and quantitative components adequately interpreted? 5.4, Are divergences and inconsistencies between quantitative and qualitative results adequately addressed? 5.5, Do the different components of the study adhere to the quality criteria of each tradition of the methods involved. Each study is evaluated against these criteria with ‘1’ indicating the study meets the criterion, ‘0’ indicating it does not, and ‘0.5’ indicating that you cannot tell

Quantitative studies relied heavily on secondary datasets, routine monitoring, or descriptive statistics, which introduced potential selection and measurement biases. Sampling strategies were frequently underreported, raising concerns about the representativeness of the data [29, 45]. Furthermore, the lack of standardised tools for measuring outcomes undermines the comparability of results [34, 39]. Studies relying on self-reported data (e.g., surveys) were particularly prone to recall bias and subjectivity [18, 40].

Mixed-methods studies demonstrated considerable variability in the integration of qualitative and quantitative findings. While some studies successfully synthesised diverse data sources to draw robust conclusions [21, 38], others fell short of systematically aligning their findings [44]. This limitation highlights the challenges of methodological integration and the potential for bias in interpretation.

The prevalence of ‘Can’t tell’ responses in the MMAT assessment, especially in qualitative and mixed-methods studies, posed challenges in evaluating methodological quality. These scores typically reflected unclear or absent reporting of study design, data collection, or analytical methods. To manage this variability, we did not exclude studies based on MMAT score alone; however, studies with limited methodological clarity were down-weighted during synthesis. This pragmatic approach ensured that studies with stronger design contributed more substantially to the proposed IN framework, while still capturing insights from a diverse evidence base.

Overall, most studies demonstrated some methodological limitations, particularly in reporting completeness and potential biases in data interpretation, though they still provided valuable insights into IN models. Common issues included the reliance on anecdotal or secondary data, insufficient detail on data collection protocols, and limited consideration of methodological limitations. While the studies provide rich insights into the effectiveness of integrated neighbourhoods and related interventions, the variability in methodological rigour underscores the need for standardised frameworks to improve reliability and validity in future research.

## Discussion

This systematic review consolidates the diversity in conceptualisation and operationalisation of IN models, emphasising their potential to address complex health and social needs through community-centred, cross-sector collaborations. Across the studies reviewed, INs demonstrated a shared goal of integrating health, social care, and other community resources to improve health outcomes, reduce health inequalities, and address broader social determinants of health. However, the mechanisms and focus areas within each model were tailored to specific population needs, reflecting significant variability in leadership structures, workforce integration, and engagement methodologies. Overall, seven key domains emerged from this synthesis, forming a proposed framework for understanding IN models: the integrator host, integrator enablers, integrator partnership principles, core integrated workforce, core areas of work, integrated services provided and workforce delivering integrated care. These domains offer a structured lens through which to examine the functioning and impact of INs.

A prominent theme identified is the emphasis on community empowerment and participatory approaches in IN models. Certain models promoted a shift toward community-driven healthcare, leveraging local leadership to foster preventive care and address social determinants in underserved areas [19], whilst others used creative, non-clinical interventions to enhance mental well-being, exemplifying the potential of arts-based IN models to support mental health and social inclusion for isolated individuals [17]. This diversity in approaches reflects the adaptability of IN models to address varying community needs through both traditional and innovative means. Evaluation and impact assessment approaches among IN models were equally varied, with many relying on informal feedback evaluations.

Barriers and facilitators to IN success were also identified. Common barriers included funding constraints, digital exclusion, and organisational resistance to change, while facilitators of success included strong local leadership, community engagement, and cross-sector collaboration. Notably, models that incorporated a flexible, diverse workforce, comprising community health champions, wellness coaches, and cultural liaisons, demonstrated enhanced responsiveness to community needs, reinforcing the importance of adaptable workforce structures in IN models. Additionally, hybrid service models that blend digital and in-person engagement were particularly effective in maintaining accessibility during the COVID- 19 pandemic, a key finding for future IN resilience planning [20].

Despite the importance of our work in conducting a comprehensive search across multiple databases with the inclusion of grey literature, a series of limitations are to be mentioned. Firstly, the heterogeneity across included studies represents a significant limitation. The conceptualisation and operationalisation of INs varied widely. This variability in IN design, population focus, and service scope introduces challenges in drawing generalised conclusions, as each model has unique features tailored to specific community needs and local contexts. The lack of a standardised IN framework complicates the aggregation and comparison of findings, underscoring a need for more cohesive definitions and typologies for IN models in future research. Secondly, most studies assessed outcomes through informal or subjective metrics, such as participant feedback. Whilst these informal metrics are valuable, without consistent quantitative measures, it is difficult to reliably compare outcomes across IN models. Many studies exhibited significant risk of bias, as discussed previously. Furthermore, most studies lacked long-term follow-up data, making it hard to assess the sustainability of these models. Additionally, there may be similar models internationally under different names that our study did not identify. While the final sample was predominantly composed of UK-based studies, this may reflect a regional concentration of IN initiatives, although international models were included where available (e.g., Canada, Germany, USA), it must be recognised as a limitation. Lastly, contextual factors like socioeconomic conditions, policy support, and digital access were inconsistently addressed across studies. Barriers such as funding constraints and digital exclusion, commonly cited in studies, likely vary by setting, limiting the applicability of results to different regions with added difficulty of transferring the results to other countries [20, 42].

In shaping future directions for IN models, providers, policymakers and academics have an opportunity to build upon the key domains identified in this systematic review. These domains — *integrator host, integrator enablers, integrated partnerships, core integrated workforce, core areas of work, integrated services provided, and workforce delivering integrated care* — provide a structured framework for advancing IN effectiveness and addressing diverse community health needs and should be considered as a starting point when developing an IN model. This framework compliments the national drivers for INs, such as the Fuller report and the Darzi report—both support more integration through strong leadership and coordination, but do not offer a clear operational plan [8, 47].

For provider hosts, such as a PCN or a Trust, the framework outlines roles and responsibilities. The *host* being the central coordination point, enabling smoother collaboration and resource allocation across sectors. The host is underpinned by dedicated *integrator enablers and workforce*, which will enable high functioning *integrated* teams and coordination of care. By focusing and identifying *core work areas,* the INs can deliver person centred care across the health and wellbeing spectrum and shift the dial of care from reactive to proactive, as well as addressing gaps and inequalities. Connecting the right workforce from healthcare professionals, social care staff, and community-based roles (e.g., community health champions)—INs can personalise and tailor their work to service their communities.

For Policymakers, Commissioners, and Integrated Care Boards (ICBs)—the framework gives clarity in commissioning INs in a way that is understood by all providers (statutory and nonstatutory). For commissioners, investment and structural support are where they can be key drivers. Supporting integrator hosts, such as community-led health bodies or regional NHS trusts, can enhance leadership stability and provide a foundation for coordinated IN operations. Investing in integrator enablers, including digital infrastructure and policy initiatives that facilitate cross-sector partnerships, will ensure that IN models remain accessible and adaptable. Policymakers should encourage integrated partnerships across sectors and emphasise the importance of collaborative workforces within INs. Strengthening core areas of work that address health inequities through preventative and supportive services and expanding the services provided can support INs in meeting broad health and social needs effectively. The outcomes and impact of such models can be quality assured, improved, compared with other areas or sites in a consistent way.

For academics, these domains highlight specific research opportunities. The framework provides a clear structure for researchers to investigate various aspects of INs, from the effectiveness of integrator enablers to the impact of integrated services. Specific metrics and methodologies to evaluate each domain, such as host attributes or integrator enablers, could support more standardised evaluation across IN models, hence resulting in better contribution to the evidence base for integrated care models. Future studies can investigate the effectiveness of different integrator enablers in overcoming common barriers, such as digital exclusion or funding constraints, and assess how each integrated partnership—whether with local councils, social care, or community organisations—impacts health outcomes. Academics can also focus on longitudinal studies that analyse the sustainability of the core integrated workforce and measure the long-term impact of core areas of work in addressing health inequities. These frameworks could be useful in identifying best practices and scaling IN approaches.

In conclusion, this work highlights the heterogenous body of literature surrounding IN models but showcases the potential of IN models to strengthen community health by integrating health, social care, and local resources. The proposed framework provides a standardised approach to guide the development and evaluation of INs; and can be used as a starting point in supporting their scalability and effectiveness. By doing so, it aims to equip healthcare leaders and policymakers with the tools needed to implement more effective, sustainable, and equitable neighbourhood-based care. The framework may provide a benchmark for evaluating INs, informing both future research and the development of national and international healthcare strategies.

## Data Availability

The datasets used and/or analysed during the current study are available from the corresponding author on reasonable request.

## Abbreviations

IN: Integrated neighbourhoods
MMAT: Mixed methods appraisal tool
PCN: Primary care networks
PMN: Patient’s medical neighbourhood
PRISMA: Preferred Reporting Items for Systematic Review and Meta-Analysis
HN: Healthcare neighbourhood
NHS: National Health Service
ABCD: Asset-Based Community Development
CCG: Clinical Commissioning Group

## References

[1] Integrated care systems explained. The King’s Fund; 2021. https://www.kingsfund.org.uk/publications/integrated-care-systems-explained. Accessed 28 Jul 2022.

[2] Goddard M. Integrated care systems and equity: prospects and plans. J Integr Care. 2023;31:29–42.

[3] NHS Confederation. Neighbourhood integration project. https://www.nhsconfed.org/networks-countries/community-network/neighbourhood-integration-project. Accessed 1 Oct 2024.

[4] Bosdijk A, Nieboer AP, Cramm JM. The development of an integrated neighborhood approach for health promotion and prevention: a qualitative exploration of stakeholders’ views. Health Res Policy Syst. 2023;21:125. 38017576 10.1186/s12961-023-01077-4 PMC10683097

[5] Leichsenring K. Developing integrated health and social care services for older persons in Europe. Int J Integr Care. 2004;4: e10. 16773149 10.5334/ijic.107 PMC1393267

[6] Sandhu S, Sharma A, Cholera R, Bettger JP. Integrated health and social care in the United States: a decade of policy progress. Int J Integr Care. 2021;21:9. 34785994 10.5334/ijic.5687 PMC8570194

[7] Community network. neighbourhood integration project. delivering integrated care at neighbourhood level. approaches to workforce. 2020. https://www.nhsconfed.org/system/files/2021-06/Delivering-integrated-care-at-neighbourhood-level-approaches-to-workforce.pdf. Accessed 1 Oct 2024.

[8] NHS England. Next steps for integrating primary care: fuller stocktake report. NHS England. Website: https://www.england.nhs.uk/publication/next-steps-for-integrating-primary-care-fuller-stocktake-report/. Accessed 2 Oct 2024.

[9] NHS England. Primary care networks (PCNs). https://www.england.nhs.uk/long-read/primary-care-networks-pcns/. Accessed 17 Dec 2024.

[10] Nuffield Trust. Integrated neighbourhood teams: lessons from a decade of integration. Nuffield Trust. https://www.nuffieldtrust.org.uk/news-item/integrated-neighbourhood-teams-lessons-from-a-decade-of-integration. Accessed 1 Oct 2024.

[11] College of Family Physicians of Canada. Best advice guide: patient’s medical neighbourhood. Mississauga: College of Family Physicians of Canada; 2020. p. 15.

[12] Moher D, Liberati A, Tetzlaff J, Altman DG, Group P. Preferred reporting items for systematic reviews and meta-analyses: the PRISMA statement. Int J Surg. 2010;8:336–41. 20171303 10.1016/j.ijsu.2010.02.007

[13] Covidence systematic review software. Melbourne: Veritas Health Innovation; 2019. Available at www.covidence.org.

[14] Campbell M, McKenzie JE, Sowden A, Katikireddi SV, Brennan SE, Ellis S, et al. Synthesis without meta-analysis (SWiM) in systematic reviews: reporting guideline. BMJ. 2020;368:l6890. 31948937 10.1136/bmj.l6890 PMC7190266

[15] Clarke V, Braun V. Thematic analysis. J Posit Psychol. 2017;12:297–8.

[16] Hong QN, Fàbregues S, Bartlett G, Boardman F, Cargo M, Dagenais P, et al. The mixed methods appraisal tool (MMAT) version 2018 for information professionals and researchers. EFI. 2018;34:285–91.

[17] Bungay H, Clift S. Arts on prescription: a review of practice in the U.K. Perspect Public Health. 2010;130:277–81. 21213564 10.1177/1757913910384050

[18] Healthwatch Enfield. Community powered Edmonton: report. 2022.

[19] Lent A, Pollard G, Studdert J. A community-powered NHS making prevention a reality. New Local; 2022. Website: https://www.newlocal.org.uk/publications/community-powered-nhs/. Accessed 2 Oct 2024.

[20] Health and Social Care Alliance Scotland. Learning from changes to social care during COVID-19. ALLIANCE; 2021. Website: https://www.alliance-scotland.org.uk/wp-content/uploads/2021/12/Learning-from-changes-to-social-care-during-COVID-19.pdf. Accessed 1 Oct 2024.

[21] Dickerson J, Moss R, Hou B, Kelly B, Reece S, Mohammed M, et al. The Bradford inequalities research unit: final report. Website: https://borninbradford.nhs.uk/wp-content/uploads/2024/11/BIRU-Final-Report_v2.0_Jan24.pdf. Accessed 2 Oct 2024.

[22] Such E, Walton E, Delaney B, Harris J, Salway S. Adapting primary care for new migrants: a formative assessment. BJGP Open. 2017;1:1. 10.3399/bjgpopen17X100701 PMC6172675 30564648

[23] South J, Southby K, Freeman C, Bagnall AM, Pennington A, Corcoran R. Community wellbeing case study synthesis. London: What Works Centre for Wellbeing; 2021.

[24] Local Government Association. A glass half-full: 10 years on review. 2020.

[25] Simpson M. Health creation: how can primary care networks succeed in reducing health inequalities? Royal College of General Practitioners Health Inequalities Standing Group; 2021. https://thehealthcreationalliance.org/wp-content/uploads/2021/02/PCNs-workshop-series-report-FINAL-_-2-February-2021-.pdf. Accessed 2 Oct 2024.

[26] Lavans A, Jenkins B, Jesurasa A. Social prescribing case studies: full report. Public Health Wales; 2023. Website: https://phw.nhs.wales/services-and-teams/primary-care-division/social-prescribing/social-prescribing/social-prescribing-case-studies-full-report/. Accessed 1 Oct 2.

[27] Jones C, Robertson N, Kinneret L, Coolet S. Social prescribing in the East Midlands. In: 2019 Survey results and regional case studies. University of Leicester; 2019. Website: https://healthinnovation-em.org.uk/images/Social_Prescribing_in_the_East_Midlands_-_2019_survey_findings_and_case_studies.pdf. Accessed 2 Oct 2024.

[28] Tull K. Vaccine hesitancy: guidance and interventions. 2024.

[29] Kassianos AP, Ignatowicz A, Greenfield G, Majeed A, Car J, Pappas Y. “Partners rather than just providers…”: a qualitative study on health care professionals’ views on implementation of multidisciplinary group meetings in the north west London integrated care pilot. IntJ Integr Care. 2015;15:e032. 26351410 10.5334/ijic.2019 PMC4560079

[30] Jackson C, Manley K, Vibhuti M. Change starts with me: an impact evaluation of a multiprofessional leadership programme to support primary care networks in the South East of England. Leadersh Health Serv. 2022;35:309–37.

[31] Public Health England. Whole systems approach to obesity programme: learning report. Public Health England; 2019. Website: https://www.gov.uk/government/publications/whole-systems-approach-to-obesity. Accessed 2 Oct 2024.

[32] Future Spaces Foundation. Kinship in the City. Website: https://www.housinglin.org.uk/Topics/type/Kinship-in-the-City-Urbanloneliness-and-the-Built-Environment/#:~:text=This%20report%20by%20Future%20Spaces,environment%20to%20improve%20social%20cohesion. Accessed 2 Oct 2024.

[33] Cheetham M, Van der Graaf P, Khazaeli B, Gibson E, Wiseman A, Rushmer R. “It was the whole picture” a mixed methods study of successful components in an integrated wellness service in North East England. BMC Health Serv Res. 2018;18:200. 29566687 10.1186/s12913-018-3007-z PMC5863899

[34] Reynolds J. Boundary work: understanding enactments of ‘community’ in an area-based, empowerment initiative. Crit Public Health. 2018;28:201–12.

[35] Orton L, Halliday E, Collins M, Egan M, Lewis S, Ponsford R, et al. Putting context centre stage: evidence from a systems evaluation of an area based empowerment initiative in England. Crit Public Health. 2017;27:477–89.

[36] Findlay G, Tobi P. Well communities. Perspect Public Health. 2017;137:17–20. 28074690 10.1177/1757913916680329

[37] The Health Foundation. Making it happen. In: Practical learning and tips from the five realising the value local partner sites. The Health Foundation; 2016. Website: https://www.health.org.uk/reports-and-analysis/reports/making-it-happen. Accessed 2 Oct 2024.

[38] European Observatory on Health Systems and Policies, Groene O, Hildebrandt H, Ferrer L, Stein KV. People-centred population health management in Germany. Eurohealth. 2016;22:7–10.

[39] Farmer J, Nimegeer A. Community participation to design rural primary healthcare services. BMC Health Serv Res. 2014;14:130. 24649834 10.1186/1472-6963-14-130 PMC3999926

[40] Collins B. Intentional whole health system redesign. Southcentral Foundation’s’ Nuka’system of care. London: The King’s Fund; 2015.

[41] National Institute for Health and Care Research (NIHR). Putting community health workers at the heart of primary care. 2021. https://www.arc-nwl.nihr.ac.uk/news/putting-community-health-workers-at-the-heart-of-primary-care. Accessed 15 Oct 2024.

[42] NHS Confederation. Combatting loneliness in York. 2022. https://www.nhsconfed.org/case-studies/combatting-loneliness-york. Accessed 15 Oct 2024.

[43] Morgan J. Emergency department costs averted attributed to community health centres in Ontario. 2023.

[44] NHS England. Working together to improve health in Fleetwood. 2023. https://www.england.nhs.uk/publication/working-together-to-improve-health-in-fleetwood/. Accessed 15 Oct 2024.

[45] Institute of Health Equity. Marmot review 10 years on. 2020. Website: https://www.instituteofhealthequity.org/resources-reports/marmot-review-10-years-on. Accessed 2 Oct 2024.

[46] Curry N, Harris M, Gunn L, Pappas Y, Blunt I, Soljak M, et al. Integrated care pilot in north west London: a mixed methods evaluation. Int J Integr Care. 2013;13:e027. 24167455 10.5334/ijic.1149 PMC3807631

[47] Darzi A. Independent investigation of the NHS in England. 2024. 10.1136/bmj.q1676 39059999

